# Editorial: Precision Medicine in Obstetrics: Pregnancy Complication

**DOI:** 10.3390/jpm13020305

**Published:** 2023-02-10

**Authors:** Serena Bertozzi, Bruna Corradetti, Arrigo Fruscalzo, Ambrogio P. Londero

**Affiliations:** 1 Department of Surgery, Breast Unit, DAME, University Hospital of “Santa Maria della Misericordia”, 33100 Udine, Italy; 2 Ennergi Research (Non-Profit Organisation), 33050 Lestizza, Italy; 3 Center for Precision Environmental Health, Baylor College of Medicine, Houston, TX 77030, USA; 4 Clinic of Obstetrics and Gynecology, University Hospital of Fribourg, 79106 Fribourg, Switzerland; 5 Department of Neuroscience, Rehabilitation, Ophthalmology, Genetics, Maternal and Infant Health, University of Genoa, 16132 Genova, Italy; 6 Obstetrics and Gynecology Unit, IRCCS Istituto Giannina Gaslini, 16147 Genova, Italy

Millions of women give birth every year worldwide. Despite technological advances in diagnosis and monitoring, a considerable number of women are still experiencing pregnancy and childbirth complications (e.g., preterm delivery, pre-eclampsia, fetal growth restriction, etc.). The social and healthcare implications associated with these complications make them a significant public health concern. Moreover, the progressive delay in childbearing and its negative association with pregnancy outcomes due to cultural, social, and economic changes has led to the onset of more frequent fertility problems. Due to the multifactorial etiology of these complications, with genetic-, environment-, and lifestyle-associated factors being involved, developing methods able to overcome them during gestation is a high priority. 

Precision medicine represents a bold research area with the potential to revolutionize the way we approach and treat diseases. Alongside nanotechnology and molecular medicine, precision medicine enables the specific delivery of therapeutic agents to cells and organs of interest, thus improving treatment outcomes and reducing side effects. In the obstetric field, several opportunities exist to leverage precision medicine as a diagnostic and treatment tool, as well as for the development of innovative strategies with the potential to overcome the challenges associated with fetal growth restriction, preterm birth, and fetal abnormalities, to state a few. The present collection highlights the role of precision medicine in obstetrics and includes preclinical studies testing the early diagnostic, preventive, and therapeutic potential of a tailorable, patient- and disease-specific approach to maternal–fetal pathologies. 

Compared to the traditional “one-size-fits-all” strategy, a more specific and targeted approach offers the opportunity for safer and more effective personalized treatments, with improved outcomes and a reduced risk of side effects, which consequently lead patients to a better quality of life and even longer survival [1]. Bertozzi et al. [2] provide an extensive overview of the application of personalized medicine to obstetrics, focusing on its current applications to overcoming issues currently associated with managing pregnancy-related pathologies (including pre-eclampsia, diabetes, and cancer) and offering insights on the potential role these may play in developing intervention options. The authors also discuss the potential toxicity associated with nanotechnology-based approaches and the difficulties of some of them in crossing the placental barrier, and propose exosomes as a natural nanoscopic alternative with inherent targeting specificity and molecular moieties. The studies considered in this review also shed light on the need to establish a universal set of guidelines for the quality control of nanomaterials used in pregnancy by regulatory agencies in order to allow pregnant women to safely reap the benefits of nanotechnology-enabled products while assisting in the implementation of exposure controls to ensure maternal and fetal safety. Dieste-Pérez et al. proposed a personalized model to predict small for gestational age (SGA) newborns at delivery using fetal biometrics, maternal characteristics, and pregnancy biomarkers [3]. In a retrospective cohort study of 12,912 cases, the authors compared the potential value of third trimester screening, based on estimated weight percentile, using universal ultrasound at 35–37 weeks of gestation, with a combined model integrating maternal characteristics and biochemical markers (PAPP-A and β-HCG) for the prediction of SGA newborns. Their analysis revealed contingent screening models as being more sensitive than third trimester ultrasound screening, when used as the sole technique for predicting SGA at delivery. 

Laboratory and radiological investigations supported by precision medicine in the field of early diagnosis include biomarkers [4], risk scores [5,6], cardiotocography [7], and magnetic resonance imaging (MRI) [8] for the early detection of potentially critical conditions during pregnancy. MRI is a reproducible diagnostic imaging technique for the evaluation of a wide variety of pathologies that offers several advantages compared to approaches that are operator-dependent or expose patients to ionizing radiations. As such, MRI is commonly used in pregnant women to evaluate, most frequently, acute abdominal and pelvic pain or placental abnormalities, as well as neurological or fetal abnormalities, infections, or neoplasms. Gatta et al. discuss the potential adverse effects of MRI performed during pregnancy due to the administration of gadolinium-based contrast agents (GBCAs), which are able to cross the placental barrier and potentially harm the health of the mother and the fetus [8]. Their review focuses on the effects of contrast and non-contrast MRI use during pregnancy. Based on the data obtained from the literature, the consensus is that GBCAs are linked to little or no risk for the mother, but results on the safety profile for the fetus are less conclusive. In preclinical studies, these authors have shown that nanotechnology-based formulations of gadolinium (liposomal nanoparticle-based blood-pool gadolinium contrast agents) represent a safer alternative as they can circulate without penetrating the placental barrier and, therefore, do not expose the fetus to the contrast agent during pregnancy, preserving it from any potential risks. 

Among gestational complications, diabetes mellitus (GDM) adversely affects maternal and offspring health. GDM is commonly associated with a variety of risk factors, such as body mass index and age. Emerging data, however, suggest a multifactorial etiology of GDM, with both genetic and environmental components playing a role. Perišić et al. contributed to this collection by presenting a recently developed polygenic risk score for GDM to investigate relationships between its genetic architecture and genetically constructed risk factors and biomarkers [6]. Their results demonstrate that the polygenic risk score can be used as an early screening tool that identifies women at higher risk of GDM before its onset, allowing comprehensive monitoring and preventative programs to mitigate the risks. Through a machine learning approach applied to case–control genetics datasets, the same authors also demonstrated that a risk score can also be used as an early screening test for gestational hypertensive disorders (GHDs) and pre-eclampsia [5]. In all cases, the polygenic risk score coupled with other known risk factors and maternal medical history showed promise in the identification of women at higher risk of pregnancy-associated complications before their onset and enabled the stratification of patients into low-risk and high-risk groups for monitoring and preventative programs. In the attempt to identify biomarkers for the detection of GDM, Fruscalzo et al. revealed an altered expression and staining pattern of retinoic acid (STRA6) in the placental tissue of the pregnancies affected by GDM compared to the controls [4]. According to the authors, these findings indicate an impairment of the retinoid pathway in the context of GDM, regarded as a common pathology of pregnancy, and data obtained could help to direct future investigations to improve our understanding of the disease pathogenesis and define new approaches for precision medicine. 

Precision medicine is gaining momentum in the obstetrics field for its potential to revolutionize the standard of care and lead to better outcomes for both mothers and babies. Precision-medicine-based strategies have proved to be effective for therapeutic applications, due to their ability to be tailored based on individual specific requirements, and safer compared to established diagnostic tools. The possibility of improving prevention and containing side effects will contribute to controlling overall healthcare costs through the early diagnosis of selected high-risk patients and the treatment of potentially critical conditions for pregnant women and their offspring. Precision medicine is not free from concerns. It comes with limited accessibility, especially in low-middle-income countries, which notoriously suffer from an unequal distribution in healthcare. Furthermore, as a relatively new approach, other concerns exist about its long-term effects and the lack of standardization, which in turn could undermine the comparison of outcomes in determining the most effective treatment [2,9]. In addition, the environmental impact of the production and disposal of nanomaterials is not neglectable [10], and neither are the ethical concerns surrounding genetic information management in medical care and potential discrimination. The promise and the limitations of precision medicine have been comprehensively reported in the Special Issue, along with the invitation to clinicians and scientists to keep contributing to the fields of precision medicine and obstetrics with additional research, which will be extremely beneficial in guiding the future developments of personalized diagnostic techniques and treatments.

## Data Availability

Not applicable.
